# Machine Learning-Based Prediction of Ablation Groove Geometry and Heat-Affected Zone Formation in Femtosecond Laser Micromachining of Aluminum

**DOI:** 10.3390/ma19143028

**Published:** 2026-07-14

**Authors:** Mateusz Tański, Robert Barbucha, Marek Kocik, Todor Petrov, Mostefa Mohamed-Seghir

**Affiliations:** 1 Institute of Fluid Flow Machinery, Polish Academy of Sciences, Fiszera 14, 80-231 Gdansk, Poland; brobert@imp.gda.pl (R.B.); kocik@imp.gda.pl (M.K.); 2 Faculty of Applied Mathematics and Informatics, Technical University-Sofia, 8 Kl. Ohridski Blvd., 1000 Sofia, Bulgaria; petrovts@tu-sofia.bg; 3 Department of Renewable Energy Sources and Electromobility, Gdynia Maritime University, 81-225 Gdynia, Poland; m.mohamed-seghir@we.umg.edu.pl

**Keywords:** laser processing, femtosecond laser, micromachining, artificial neural network

## Abstract

This study presents an Artificial Neural Network (ANN) approach for predicting laser-induced material modifications during femtosecond laser micromachining of aluminum. Experimental investigations were carried out to determine the influence of the average laser power and scanning speed on the width of the ablation groove and the size of the optically determined surface-discoloration width used as a proxy for the Heat-Affected Zone (HAZ). The collected dataset, consisting of 100 samples, was used to develop, train, validate, and test an ANN predictive model with two inputs, two outputs, and two hidden layers. Despite its simplicity and the relatively small dataset, the developed model achieved relatively good prediction accuracy, with an overall correlation coefficient (R) of approximately 0.95 on the test dataset. The predicted values showed reasonable agreement with the experimental results, indicating that the ANN approximated the relationship between laser processing parameters and the resulting material modifications. The presented methodology may provide a useful tool for predicting surface morphology changes and thermal effects in femtosecond laser processing of aluminum.

## 1. Introduction

Laser micromachining is an advanced manufacturing technique which utilizes laser radiation to selectively remove fine fragments of the material in order to form features with micrometer precision [1]. This technology has found widespread industrial application in the fabrication of electronic components [2], the structuring of optical elements [3,4], the production of photovoltaic cells [5,6], and the processing of other advanced materials [7,8,9].

Recent developments in laser technology and the introduction of ultrafast femtosecond lasers to the market have significantly improved the quality of laser micromachining. In contrast to the earlier technologies based on nanosecond and longer laser pulses, femtosecond laser micromachining limits the heat diffusion from the irradiated region into the surrounding material, improving the ablation efficiency and reducing the thermal degradation of the material, including the HAZ formation [10,11,12]. However, femtosecond laser ablation is a complex process, involving numerous phenomena, such as non-linear absorption, heat transfer, phase transitions, plasma formation and material ejection occurring simultaneously [13,14]. Thus, achieving a non-thermal ablation (also called cold ablation) and HAZ-free processing remains challenging and requires precise adjustment of multiple process parameters, which in turn demands understanding of the underlying ablation mechanism. Although extensive efforts have been devoted to developing analytical and numerical models of femtosecond laser–matter interaction, such approaches still face significant limitations [15,16,17], while experimental investigations remain tedious and time-consuming work. In this context, machine learning (ML) has recently emerged as a promising alternative for studying and optimizing laser micromachining processes [18,19,20].

Pioneering applications of ML in laser micromachining date back to the early 2000s. Yousef et al. [21] were among the first to propose ML as an alternative to a traditional trial-and-error approach for selecting laser parameters and showed that ANNs can successfully predict optimal drilling parameters. Later, McDonnell et al. [22] applied ML to multi-parameter optimization of the laser drilling process, showing that ML can predict complex physical phenomena and can reduce the amount of experimental data required to achieve accurate predictions compared to conventional analytical methods. Similar conclusions were drawn by Rahimi et al. [23], who modeled the geometry of Al-SiC engraving with nanosecond laser pulses. They demonstrated that, under the right conditions, ANN models can give more accurate results than a traditional analytical model based on response surface methodology. Following this, a more systematic comparison of ML algorithms was carried out by Teixidor et al. [24], who evaluated k-Nearest Neighbors, ANN, decision trees, and linear regression models for predicting the geometry of features produced by nanosecond laser milling. Their results indicated that ANNs provided the best performance in terms of dimensional accuracy of the machined features. Ding et al. [25] used ML for optimization of infrared mirror fabrication for aerospace applications and demonstrated that this approach provides accurate prediction and optimization of microstructures, resulting in improved optical performance.

When exploring the application of ML for laser micromachining, several authors have emphasized challenges related to limited training datasets and model complexity. However, Tsai et al. [26] demonstrated that a relatively simple ANN model trained using only 27 experimental data points can be successfully applied to predict the cutting quality during laser micromachining of Quad Flat Non-lead (QFN) packages. Similarly, Keerthi et al. [27] developed an ANN model to predict the circularity and HAZ size in nanosecond laser drilling, achieving high prediction accuracy despite a small training dataset also consisting of 27 entries. On the other hand, Solati et al. [28] showed that the HAZ and bearing strength in laser drilling could be effectively predicted using a simple ANN with a single hidden layer containing only 5–9 neurons.

More recently, ML has also been successfully applied to ultrafast laser micromachining. However, most ML-based investigations in ultrafast micromachining have focused predominantly on laser drilling. For example, Tani et al. [29] showed that ANNs can accurately simulate femtosecond laser drilling. They noticed that a model trained on a single-shot dataset was capable of extrapolating to multi-shot predictions. Shimahara et al. [30] applied deep learning to optimize ultrafast hole drilling with reduced power consumption. Kim et al. [31] developed ANN models to predict hole uniformity in femtosecond laser drilling. Similar studies focused on femtosecond laser drilling were also reported by other research groups [32,33,34,35].

Although the application of ML to femtosecond laser drilling has become an active research subject, femtosecond laser cutting and groove formation, involving continuous material removal and more complex thermal effects, remain practically unexplored. Moreover, most previous studies have emphasized ablation efficiency or geometrical accuracy while largely neglecting the thermal degradation of the material. In particular, the prediction of the HAZ size remains insufficiently explored, although its minimization is one of the key advantages of ultrafast micromachining.

In this paper, building on our previous work [36], we aim to address existing knowledge gaps. We investigate the use of a machine learning model for femtosecond laser groove micromachining of aluminum 3003 under limited experimental data conditions. In contrast to most ANN-based studies in this field, which focus on drilling processes and single-output prediction, the present work considers groove formation, where both groove width and optically determined HAZ proxy width are predicted simultaneously. We examine whether a simple ANN model adequately captures the nonlinear relationship between process parameters and both output responses. The results show that the ANN approach provides reasonably good predictive performance and better performance than linear and second-order polynomial regression models. The proposed model is also compared with other machine learning models reported in the literature and trained on similarly small datasets. Prediction errors and the training process are also examined. Prediction errors may arise from measurement uncertainty associated with the irregular nature of the laser ablation process. In addition to the ANN-based analysis, the underlying physical mechanisms of laser ablation and the influence of the input parameters on the micromachining process are briefly discussed.

## 2. Materials and Methods

In our experiment, the test samples were made from 200 μm thick aluminum 3003 foil. Aluminum was selected as the test material due to its wide industrial application and availability of reference data on laser micromachining of aluminum. In addition, the relatively high surface reflectivity and thermal conductivity of aluminum (237 W/mK [37,38]) simplify optical analysis of heat propagation in this material.

This study was conducted in four main stages. In the first stage, aluminum samples were prepared and processed by creating a pattern of grooves on their surface under varying scanning speeds and average laser powers. In the second stage, the resulting grooves were measured using an optical microscope. Next, the measured data were used to prepare training and testing datasets. Finally, an ANN model was developed, trained, and tested.

### 2.1. Experimental Laser Setup

Laser processing of the aluminum samples was done using an experimental setup equipped with a Pharos femtosecond laser source from Light Conversion (Vilnius, Lithuania). The laser generated pulses with a duration of 500 fs, a repetition rate of 200 kHz, a wavelength of 1030 nm, and a maximum average power of 10 W (corresponding to a pulse energy of 50 μJ). The laser beam was guided by an XY galvanometric scanner (hurryScan from ScanLab, Puchheim, Germany) and focused onto the aluminum sample with a 100 mm telecentric F-theta lens. The setup was additionally equipped with an XYZ translation stage, which was used to position the sample in the beam focal plane. The beam was focused to approximately a 100 µm spot, resulting in a maximum pulse fluence of 0.6 J/cm^2^ on the sample surface, which is typical for femtosecond micromachining of aluminum [39,40,41].

In this study, the variable input parameters were the average laser power (which determines the laser pulse energy under the fixed repetition rate) and scanning speed (which controls the delivered laser energy per unit length). These parameters were selected because they represent the main independently controllable variables during femtosecond laser processing and directly influence the laser–material interaction and the resulting processing characteristics. Although the quality of femtosecond laser material processing depends on numerous other parameters [42], some of them, such as laser wavelength, pulse duration, beam quality, and spatial beam profile, are intrinsic to the laser source and experimental configuration and therefore remained unchanged throughout this study. Other laser and material parameters, including repetition rate, laser spot size, and some material properties, could, in principle, be adjusted. However, they were intentionally kept constant to isolate the effects of the two selected process variables (average laser power and scanning speed) on the ablation results.

### 2.2. Sample Preparation and Analysis

Prior to laser processing, the aluminum foil was cut into ten square samples of 50 mm × 50 mm and cleaned with isopropyl alcohol. Next, a test pattern of ten parallel grooves was micromachined on each sample using a femtosecond laser. The grooves were 5 mm in length, and each groove in the pattern was fabricated with a different scanning speed. Each sample was machined with a different laser power. The scanning speed was varied from 10 to 100 mm/s in 10 mm/s increments, and the laser power was varied from 1 to 10 W in 1 W increments, resulting in a dataset of 100 individual entries. Femtosecond laser power stability was better than 0.5%, and scanner speed accuracy was better than 0.2%, both of which can be considered negligible in the context of this study.

Although groove depth is an important parameter for complete characterization of the ablation structures, it was not considered in the present study, since its objective was not to provide a complete reconstruction of the ablated geometry, but rather to develop a predictive model describing the influence of key laser parameters on measurable characteristics of the processed surface. Precise determination of groove depth can be challenging using standard optical methods due to optical effects that prevent clear observation of the groove inner walls. Therefore, following approaches reported in the literature [43,44,45], the present study focuses on surface groove analysis, which can be reliably determined using conventional optical microscopy. The groove width was defined as the surface distance between opposite walls.

Alongside groove width measurements, surface discoloration around the irradiation region was analyzed as an optical indicator of thermally affected material. In this study, the term optically determined HAZ proxy width refers specifically to the total transverse width of the visible surface discoloration band, measured from one outer discoloration boundary to the opposite boundary and including the ablation groove. This parameter is used as a practical optical proxy for the surface extent of thermally affected material and should not be interpreted as a direct measurement of subsurface microstructural modification. Surface discoloration is commonly considered an optical indicator of thermal effects in the material, which may be accompanied by further phenomena such as recrystallization, phase transformations, and generation of stresses and strains [46,47,48,49,50,51], and can be easily observed on the aluminum surface using optical methods. However, it should be noted that optical surface observation alone does not allow full evaluation of subsurface thermal modification. Accordingly, unless otherwise stated, the term HAZ width used throughout the manuscript refers exclusively to this optically determined proxy. When no surface discoloration was detectable beyond the groove edge, this parameter was assigned the same value as the measured groove width.

The groove and HAZ proxy widths were measured using a Nikon Eclipse TS100 metallographic optical microscope (Tokyo, Japan). For each groove, the groove width and the optically determined HAZ proxy width were measured at ten positions distributed along the groove length. At each position, both dimensions were measured perpendicular to the groove direction. The arithmetic mean of those ten values was rounded to the nearest micrometer and was used as the target value in the ANN dataset. Unless otherwise specified, the measurement results of groove and HAZ widths are presented in micrometers.

### 2.3. ANN Model

Numerous ML models have been proposed for predicting the results of laser processing, including decision trees [24,52], random forest [53,54,55], Gaussian processes [56,57], genetic algorithms [58,59], k-nearest neighbors [24], support vector machines [60,61], and artificial neural networks [62,63]. Since several studies have suggested that ANN models typically provide the best performance in laser micromachining applications, especially when the training dataset is relatively small [24], the ANN approach was adopted in this work.

**Figure 1:**
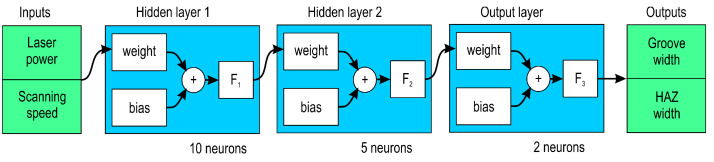
Architecture of the developed ANN model.

The ANN was constructed and implemented in MATLAB (R2021b) using the Neural Network Toolbox. It consisted of two input nodes, two hidden layers, and two output nodes. The input parameters were the average laser power and scanning speed, while the output parameters were the groove width and the HAZ proxy width. The ANN architecture was selected with the aim of achieving a balance between predictive performance and model complexity, since overly complex networks may lead to overfitting, particularly when trained on relatively small datasets [64]. During model development, several ANN configurations were evaluated by varying the number of hidden layers, the number of neurons in each layer and the transfer functions applied between the layers. The final architecture was selected based on prediction performance while maintaining a relatively simple network structure. The final ANN structure consisted of two hidden layers containing 10 and 5 neurons, respectively, as illustrated in Figure 1. The transfer functions applied in the successive layers were as follows: hyperbolic tangent sigmoid functions (F1, F2) and linear function (F3).

The dataset used for ANN training and testing was derived from our experimental measurements. Data were checked for obvious errors and prepared following commonly used data preparation guidelines [65,66]. In total, 100 data entries were collected. The dataset was randomly divided using the MATLAB “dividerand” function into three subsets: 70% for training, 15% for validation, and 15% for testing. The validation subset was used to monitor the training process and determine the early-stop point, while the test subset was not used to update the network weights and biases. It should be noted that due to the limited size and structure of the dataset, the ANN may reflect interpolation within the parameter space rather than extrapolation beyond it [67].

The network was implemented as a feed-forward backpropagation model and trained using the Levenberg–Marquardt backpropagation algorithm “trainlm” [68]. The mean squared error “mse” was used as the performance function. Prior to training, all input and output variables were normalized using the default min–max procedure. The network weights and biases were initialized using the Nguyen–Widrow procedure. Training was performed with a maximum of 1000 epochs, a minimum performance gradient of 1.0 × 10^−7^, and a maximum of six consecutive validation checks without improvement.

## 3. Results and Discussion

This section presents and discusses the experimental results of femtosecond laser micromachining of aluminum, including groove widths and HAZ proxy widths, as well as the predictions of those results obtained using the developed ANN model.

### 3.1. Experimental Results

**Figure 2:**
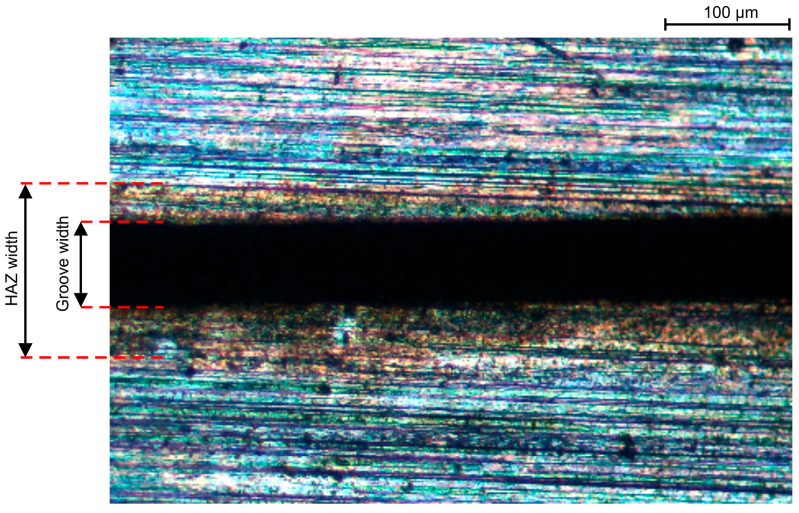
Microscopic image of the machined groove with visible surface discoloration. Groove width was 71 µm, and the optically determined HAZ proxy width, measured between the outer discoloration boundaries including the groove, was 120 µm. Laser power: 10 W, scanning speed: 80 mm/s.

Groove patterns were fabricated on aluminum samples using various combinations of laser power and scanning speed. Next, microscopic images of each groove were captured and analyzed to determine the groove and HAZ proxy widths. Figure 2 shows a typical image of the ablation groove, with clearly visible discoloration of the HAZ proxy region running along the groove length. The groove shown was produced at a laser power of 10 W and a scanning speed of 80 mm/s. The measured groove width was 71 µm, while the optically determined HAZ proxy width was 120 µm. The spatial resolution of the microscope imaging and measurement system was approximately 1 µm. For clarity, both widths are indicated in the image with arrows.

As seen in the image, the edges of the ablation groove are relatively uniform along its length, which indicates stable ablation conditions. This can be attributed to the high stability of the laser beam and a high pulse overlap of 99.6% during scanning. On the other hand, the HAZ proxy boundary is less clearly defined and more irregular. This can be explained by the uneven heat diffusion from the irradiated region, due to variations in material structure and material flow dynamics, as well as secondary thermal effects during laser ablation, such as ablation plasma heating.

**Figure 3:**
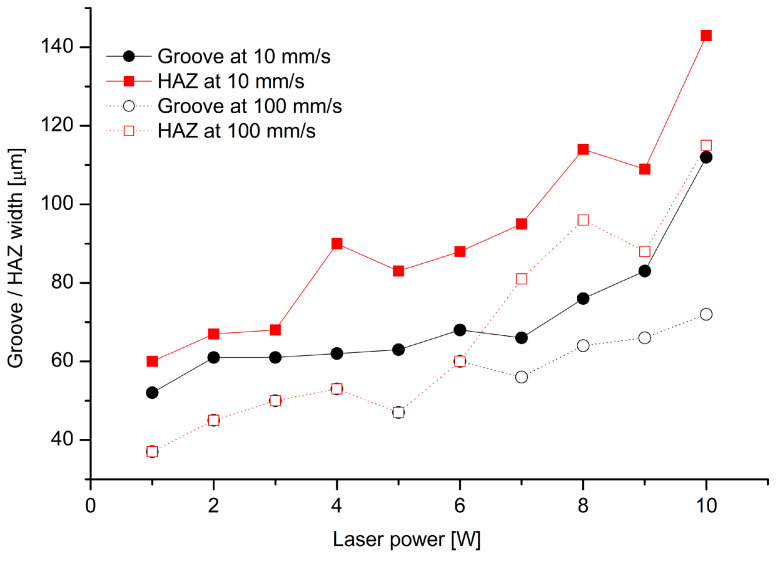
Typical examples of groove and HAZ proxy widths as a function of laser power for two values of scanning speed: 10 mm/s and 100 mm/s. The measurement points were connected by lines for plot readability.

Figure 3 shows the relationship between the groove and HAZ proxy widths and laser power for two extreme scanning speeds, 10 mm/s and 100 mm/s. The relationship between the input parameters and the resulting groove width and HAZ proxy size is nonlinear and shows substantial deviations from the general trend, which illustrates the challenges of analytical modeling of laser micromachining.

As shown in Figure 3, both groove and HAZ proxy widths generally increase with laser power, following similar trends for both scanning speeds, but the rate of increase differs in each case. At lower laser powers, the increase is moderate, whereas at higher powers, both widths significantly expand. The minimum groove width of 37 μm was obtained at a laser power of 1 W and a scanning speed of 100 mm/s. Under these process conditions, no surface discoloration was detectable beyond the groove edges (according to the adopted measurement convention, the optically determined HAZ proxy width was therefore equal to the groove width). It is worth noting that for small laser powers, groove width remained smaller than the laser spot diameter (approximately 100 μm). This can be explained by the Gaussian intensity distribution of the laser beam, where only the center of the beam exceeds the ablation threshold of aluminum, which is approximately 100 mJ/cm^2^ [69]. The remaining energy of the laser beam, as well as the residual energy from the ablation region, is dissipated into the surrounding material. At low power levels, the amount of dissipated heat is insufficient to induce significant thermal effects and to form a distinct HAZ. However, as the laser power increases, a larger fraction of the laser beam exceeds the ablation threshold, resulting in a wider ablation groove and increased heat dissipation, which leads to an increase in the HAZ width. Finally, at a laser power of 10 W, the ablation groove width reached 72 μm at a scanning speed of 100 mm/s and 112 μm at 10 mm/s. This indicates that at high laser fluence, ablation takes place not only within the region directly irradiated by the laser beam but also in the surrounding area due to intensive heat transfer. The intensive heat transfer from the irradiated region results not only in a higher ablation yield but also in the formation of an extended HAZ proxy, which reached 110 μm and 140 μm for scanning speeds of 100 mm/s and 10 mm/s, respectively.

Overall, this demonstrates that achieving the desired ablation results requires careful balancing of the process parameters, and often involves a trade-off between ablation yield and quality. This makes ML models a practical tool for predicting the results of femtosecond micromachining and for selecting the best process parameters for a specific application.

### 3.2. ANN Predictions

**Figure 4:**
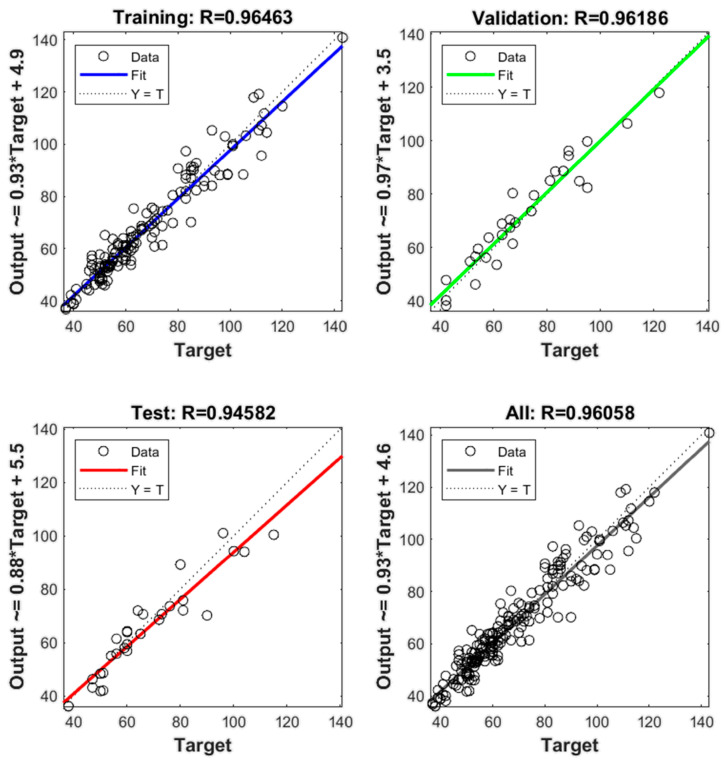
Regression plots of ANN predictions.

The performance of the trained ANN model was evaluated by comparing its predictions with the experimental data, as shown in Figure 4. The figure presents regression plots of the ANN outputs, illustrating the relationship between the predicted and target values for the training, validation, test, and combined datasets. In the case of an ideally performing algorithm, these values would be expected to lie along a straight line. However, in practice, these points deviate from a straight line, and their dispersion can be quantified using a correlation coefficient R.

For the test dataset, the overall correlation coefficient calculated for the combined outputs was approximately R = 0.95, while the coefficients for the individual outputs were R = 0.88 for groove width and R = 0.96 for the HAZ proxy width. For the validation dataset, the overall correlation coefficient was R = 0.96, while the coefficients for the individual outputs were R = 0.89 for groove width and R = 0.93 for the HAZ proxy width. For the training dataset, the overall correlation coefficient was approximately R = 0.96. The relatively high values of the correlation coefficients indicate a strong linear relationship between the predicted and target values. This indicates that the model is able to capture the underlying trend in the data and reasonably accurately predict the output values. A five-fold cross-validation analysis was additionally performed, yielding a correlation coefficient of R = 0.94 ± 0.02 and MAPE of 8.01 ± 0.84%.

To verify whether the use of the ANN was justified in terms of predictive performance rather than interpolation-driven agreement [70], the obtained results were compared with two simple models: linear regression and second-order polynomial regression, both developed using the same dataset. The linear regression model achieved an average correlation coefficient of R = 0.74 for groove width and R = 0.66 for HAZ proxy width, while the polynomial model reached R = 0.88 and R = 0.81, respectively. This shows that the polynomial model performs better than the linear model but is still less accurate than the ANN model (R = 0.95). These results suggest that the relationship between the processing parameters and both groove and HAZ proxy widths cannot be fully described by either a linear or a polynomial function, supporting the use of the ANN for this problem.

**Figure 5:**
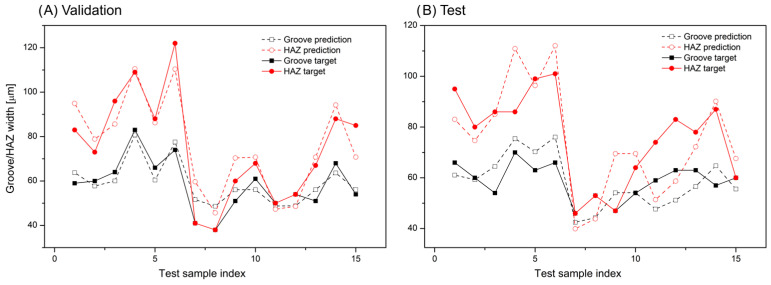
Comparison of ANN-predicted and target values for (**A**) validation dataset and (**B**) test dataset.

Despite the relatively high correlation coefficient, the deviations between predicted and target values are more clearly visible in the plots presenting their absolute values. Figure 5 illustrates the predicted and target values of the groove and HAZ proxy width for each sample in the validation and test datasets. The mean absolute percentage error for groove width prediction was approximately 11%, while for HAZ proxy width, it was approximately 15%. These values represent the overall prediction error of the ANN model within the investigated processing range and are typical for ML models trained on relatively small experimental datasets. Such error levels are generally considered acceptable in many practical applications of laser micromachining, where the final results are affected by both process fluctuations and measurement uncertainty. The residuals were analyzed with respect to both input parameters (laser power and scanning speed) for groove width and HAZ proxy width predictions. No clear systematic dependence of the prediction error on either laser power or scanning speed was observed within the investigated parameter range. The residuals were distributed both above and below zero throughout the analyzed conditions, indicating the absence of significant systematic bias. The mean signed errors were approximately −3% for the groove width and −5% for HAZ proxy width, showing only a minor tendency of the ANN model to underestimate the prediction values. Nevertheless, the overall error is influenced by a few individual data points that introduce larger deviations. It should be noted that these errors arise not only from the limitations of the ANN itself, but are also driven by the inherent variability of the laser ablation process, as discussed in Section 3.1. The reported prediction errors should be interpreted as the combined effect of model approximation error, process variability, and measurement uncertainty. However, the quantitative separation of those contributions is beyond the scope of this work.

**Figure 6:**
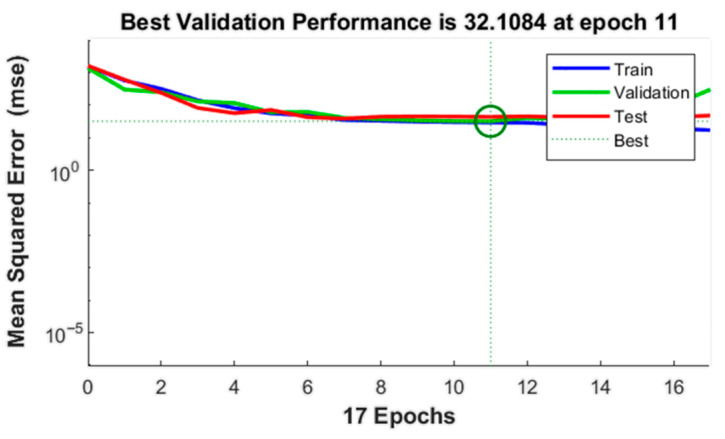
Temporal variation in MSE during ANN training and testing.

To better understand these results, a more detailed description of the ANN training process is presented below. The training progress was monitored primarily through the variation of the mean squared error (MSE) function, which represents the difference between predicted and target values (Figure 6). The MSE reached its minimum at epoch 11. Training continued until epoch 17 and was terminated after six consecutive validation checks without further improvement. The network parameters corresponding to the minimum validation MSE at epoch 11 were retained. The training, validation, and test errors were comparable, and no pronounced divergence among them was observed.

**Figure 7:**
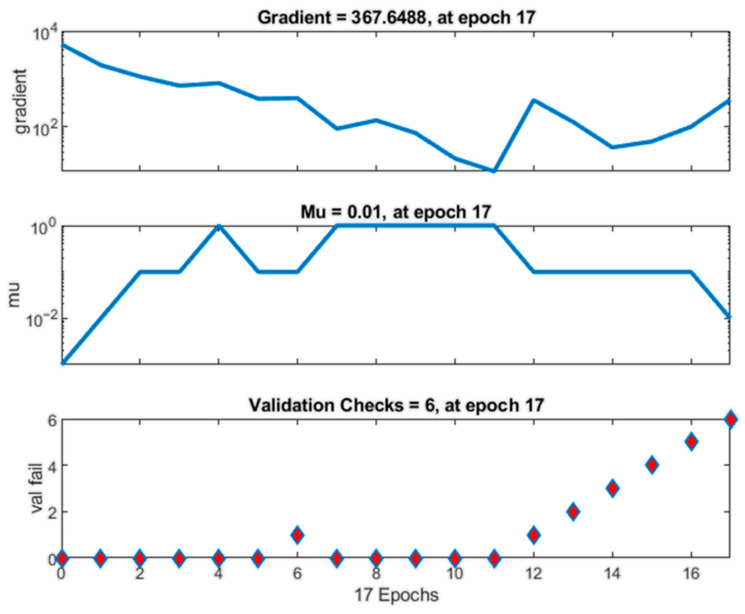
Evolution of training metrics during ANN training epochs.

The observation of the evolution of the key model parameters gives a detailed view of the ANN training process. Figure 7 presents plots of three selected model parameters: the loss function gradient, the Levenberg–Marquardt damping parameter, and the number of consecutive validation epochs without improvement.

The first plot shows the loss function gradient during training. This gradient gradually decreases during the learning process, reaching a minimum value of approximately 10 at epoch 11. As the training progresses further (up to epoch 17), the gradient begins to increase again. A stable behavior of the loss function gradient indicated that the model converges successfully and does not exhibit the gradient explosion problem. The second plot illustrates the ANN learning rate, characterized by the damping parameter of the Levenberg–Marquardt algorithm, which reflects the rate at which the weights are updated. As the training process starts, this parameter is relatively small, which indicates that the algorithm aggressively adjusts the weights. As the training progressed toward epoch 11, the damping parameter increased and stabilized at approximately 1 before decreasing during the subsequent epochs. The final plot shows the number of consecutive training epochs in which the validation error did not improve. A zero value throughout training up to epoch 11 demonstrates that each iteration (except epoch 6) improved the model and that no early stopping was triggered. The stable increase in this parameter after epoch 11 confirms that subsequent changes do not improve the model’s performance. In summary, the minimum validation error was obtained at epoch 11, while training terminated at epoch 17 after six consecutive validation checks without improvement. The model produced predictions consistent with the overall experimental trends, and gradients, weights, and parameters remained stable during training, confirming that the Levenberg–Marquardt algorithm performed effectively.

Overall, the developed ANN model is characterized by a correlation coefficient of approximately R = 0.95 and prediction errors in the range of 10–15%, which makes it a reasonably good model for practical engineering applications. It should be emphasized that this model is based on a realistic non-linear physical process, where the main challenges lie in the large variability of the outputs and the relatively limited training dataset, both of which are typical in laser micromachining optimization scenarios.

Comparing the current model with the one from our previous work [36], both models show relatively good prediction accuracy, with the previous one trained on a larger dataset achieving higher accuracy. When compared with other ANN models reported in the literature, our model is reasonably accurate [20]. However, it should be acknowledged that there are models offering higher accuracy. For example, the ANN model proposed by Tsai et al. [26], trained on a relatively small database of only 27 entries using the Levenberg–Marquardt algorithm to predict laser cutting parameters of QFN packages, exhibits an average testing error of approximately 1.5%. In that case, the high accuracy was achieved due to the limited variability of the input parameters, which effectively reduced extreme values that could influence the training process. On the other hand, a more recent model by Keerthi et al. [27], also trained using the Levenberg–Marquardt algorithm to predict laser drilling geometry and HAZ, achieved the correlation coefficient R of approximately 0.52 and 0.85 for the test dataset, respectively. These values are significantly lower than in this study, which confirms that the ablation and HAZ geometry prediction is a demanding challenge. Finally, the model proposed by Solati et al. [28], trained using a genetic algorithm to predict the geometry and HAZ of laser-drilled composite, achieved a correlation factor of approximately 0.96, only slightly higher than the one obtained in this paper. Therefore, our model performs comparably to other ANN models trained on datasets obtained from real measurements.

The obtained ANN predictions can be interpreted from the perspective of the laser–material interaction mechanism. The model indicates that both groove width and HAZ proxy strongly depend on the applied laser power and scanning speed, which is consistent with the physical nature of the femtosecond laser ablation process. The increase in laser power leads to a higher energy density delivered to the material surface, resulting in a larger volume of aluminum exceeding the ablation threshold and consequently increasing the groove width. Simultaneously, the increased energy input promotes heat transfer to the surrounding material, leading to the formation of a larger HAZ. The effect of scanning speed is related to the effective interaction time between the laser beam and the material, where lower scanning speeds increase the deposited energy per unit length and enhance both material removal and thermal effects. Therefore, the developed ANN not only reproduces the experimental trend, but also reflects the underlying relationship governing laser-induced material modification.

These results demonstrate that the ANN model can be used to estimate two output parameters simultaneously, despite its relatively simple structure and limited training dataset, while operating under high variability in both input and output parameters. The model can therefore be used to estimate groove and HAZ proxy widths during femtosecond micromachining of aluminum based on the input parameters. By predicting the expected groove geometry and HAZ proxy size for a given combination of laser power and scanning speed, the model allows the identification of process conditions corresponding to low thermal impact. This is particularly important for achieving cold ablation conditions, where material removal occurs with minimized heat accumulation and limited heat-induced modification of the surrounding material.

By using the model description provided in this paper, custom ANN models can be developed for predicting groove width and HAZ during femtosecond laser processing of aluminum, enabling easier optimization of the laser micromachining process. However, it should be emphasized that the presented model was trained using experimental data obtained for a specific material, thickness, laser system, and limited range of processing parameters. Therefore, the model should be considered primarily as a predictive tool for interpolation within the investigated parameter space, while also providing a reference point for further studies and optimization of the laser machining process. Application of the model to other materials, laser configurations, or extended processing ranges would require additional experimental validation and, if necessary, retraining of the ANN model using data representative of the new conditions.

## 4. Conclusions

This study investigates the application of an ANN model for predicting laser-induced material modifications during femtosecond laser micromachining of aluminum. Experimental investigations were performed to analyze the influence of laser power and scanning speed on the resulting ablation groove geometry and optically determined HAZ proxy. The obtained experimental data were then used to develop and evaluate an ANN-based predictive model describing the relationship between laser processing parameters and the resulting surface and thermal effects. Our main findings can be summarized as follows:

- The relationship between the input parameters (laser power, scanning speed) and the output parameters (groove and HAZ proxy size) is nonlinear, making it challenging to describe using conventional analytical models.
- The developed ANN model showed reasonable predictive performance for both groove and HAZ proxy widths, achieving an overall correlation coefficient R = 0.95 between predicted and target values for a test dataset.
- The relatively simple ANN architecture (2-10-5-2), consisting of two input nodes, two hidden layers, and two output nodes, was sufficient to approximate the observed relationship between input and output parameters.
- The model was effectively trained, validated, and tested using a limited dataset composed of only 100 experimental data entries.
- The ANN model remained stable through the training process, and no pronounced divergence between training, validation and test errors was observed.
- A source of ANN prediction error may arise from the process variability associated with the irregular nature of the laser ablation process.
- Within the investigated material and processing range, the developed ANN model may support estimation of groove and HAZ proxy widths. Its application to other conditions requires additional experimental validation.

Our future work will focus on extending the model to other materials and processing regimes and exploring its use in industrial micromachining applications.

## Data Availability

The original contributions presented in this study are included in the article. Further inquiries can be directed to the corresponding author.

## Abbreviations

The following abbreviations are used in this manuscript:

ANN	Artificial Neural Network
HAZ	Heat-Affected Zone
ML	Machine Learning
MSE	Mean Squared Error
